# A rare cause of narrow QRS complex tachycardia: the tortoise and the hare

**DOI:** 10.1007/s12471-022-01686-8

**Published:** 2022-04-29

**Authors:** S. C. Yap

**Affiliations:** Department of Cardiology, Erasmus Medical Centre, University Medical Centre Rotterdam, Rotterdam, The Netherlands

## Answer

**Fig. 1:**
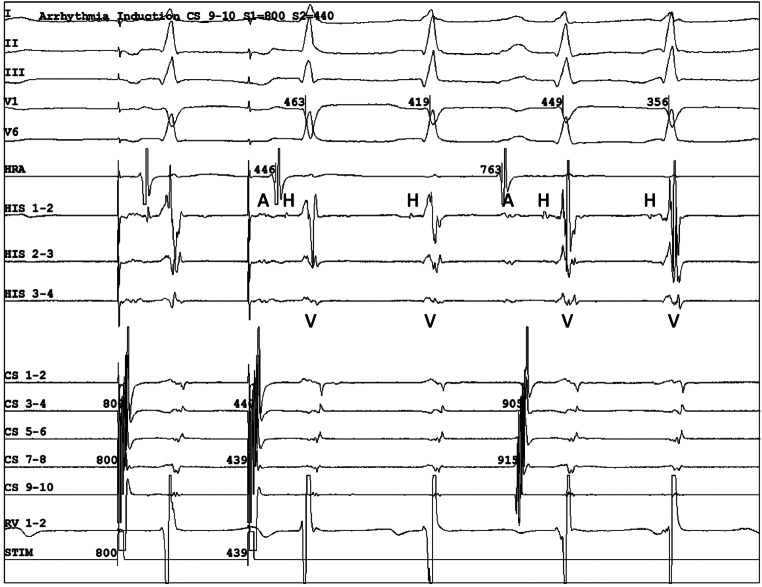
Electrophysiological study confirming ‘double firing’ phenomenon. This occurred after a programmed atrial extrastimulus and also during the subsequent sinus beat. Note that one atrial complex (A) is followed by a dual ventricular response preceded by a corresponding His potential (H). The conduction delay between the fast and slow pathway was 356 ms. *A* atrium, *CS* coronary sinus, *H* His potential, *HRA* high right atrium, *RV* right ventricle, *V* ventricle

**Fig. 2:**
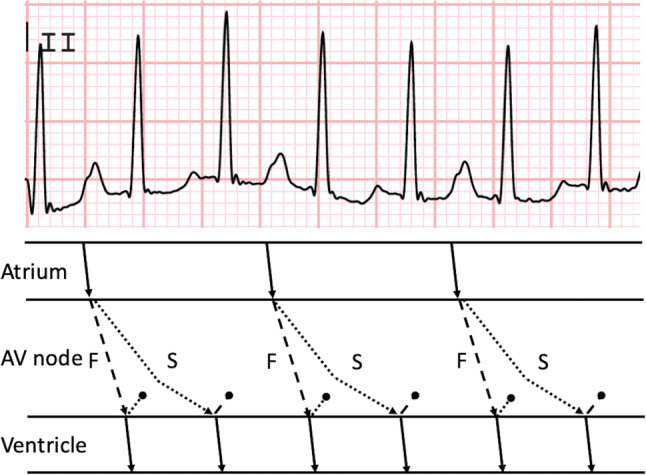
Putative mechanism of DAVNNT. **a** ECG demonstrating DAVNNT in our patient. One P wave is followed by two QRS complexes. The cycle length between P waves is 640 ms. Note that the P waves are positive in lead II ruling out a retrograde P wave; **b** Sinus beats are conducted anterogradely via the fast (dashed line) and slow (*dotted line*) pathways from the AV node. There should be a critical delay in the slow pathway to allow recovery of refractoriness in the His-Purkinje system following activation by the fast pathway. Furthermore, there is retrograde unidirectional block in both pathways (denoted by *black dot*). *F* fast pathway, *S* slow pathway

The patient underwent an electrophysiology study. At baseline, there was sinus rhythm with normal AH and HV intervals. Dual atrioventricular (AV) nodal physiology with manifest 1:2 AV conduction (‘double fire’) was observed during atrial extrastimuli (Fig. 1). During sinus acceleration, periods of sustained 1:2 AV conduction were observed. After administration of isoproterenol, a fast-slow AV nodal re-entrant tachycardia (AVNRT) could be induced with a tachycardia cycle length of 290 ms. After radiofrequency ablation of the slow pathway at the right inferoseptal area, no AH jump was present and no tachycardia could be induced. The presenting ECG was, most likely, a dual AV nodal non-re-entrant tachycardia (DAVNNT) (Fig. 2).

DAVNNT is caused by simultaneous antegrade conduction over the fast and slow pathways. DAVNNT is a rare arrhythmia and a systematic review in 2016 identified only 68 cases in the literature [1]. It is often misclassified as atrial fibrillation or premature beats. The combination of AVNRT and DAVNNT is even rarer [2]. DAVNNT may cause tachycardiomyopathy [3]. The ECG provides the clues for the diagnosis, demonstrating one normal P wave followed by two QRS complexes. These QRS complexes are usually narrow, but aberrancy is possible. Aberrant conducted QRS complexes are often misclassified as premature ventricular complexes. The differential diagnosis of AVNRT with 2:1 VA conduction is refuted by the absence of retrograde P waves (positive P‑wave morphology in inferior leads) and the slightly irregular RR intervals. Ablation of the slow pathway eliminates dual AV nodal conduction and is the cornerstone of invasive treatment.
